# Mine Clay Washing Residues as a Source for Alkali-Activated Binders

**DOI:** 10.3390/ma15010083

**Published:** 2021-12-23

**Authors:** Caterina Sgarlata, Alessandra Formia, Cristina Siligardi, Francesco Ferrari, Cristina Leonelli

**Affiliations:** 1 Department of Engineering “Enzo Ferrari”, University of Modena and Reggio Emilia, 41125 Modena, Italy; cristina.siligardi@unimore.it (C.S.); cristina.leonelli@unimore.it (C.L.); 2 Sibelco Ankerpoort NV, 6223 EP Maastricht, The Netherlands; alessandra.formia@sibelco.com; 3 Sibelco Italia S.p.A., 41053 Maranello, Italy; francesco.ferrari@sibelco.com

**Keywords:** mine clay washing residues, alkali-activated materials, geopolymers, waste, recycling, synthesis, thermal properties, mechanical properties, durability

## Abstract

The aim of this paper is to promote the use of mine clay washing residues for the preparation of alkali activated materials (AAMs). In particular, the influence of the calcination temperature of the clayey by-product on the geopolymerization process was investigated in terms of chemical stability and durability in water. The halloysitic clay, a mining by-product, has been used after calcination and mixed with an alkaline solution to form alkali activated binders. Attention was focused on the influence of the clay’s calcination treatment (450–500–600 °C) on the geopolymers’ microstructure of samples, remaining in the lower limit indicated by the literature for kaolinite or illite calcination. The mixtures of clay and alkali activators (NaOH 8M and Na-silicate) were cured at room temperature for 28 days. The influence of solid to liquid ratio in the mix formulation was also tested in terms of chemical stability measuring the pH and the ionic conductivity of the eluate after 24-h immersion time in water. The results reported values of ionic conductivity higher for samples made with untreated clay or with low temperature of calcination (≥756 mS/m) compared with values of samples made with calcined clay (292 mS/m). This result suggests that without a proper calcination of the as-received clay it was not possible to obtain 25 °C-consolidated AAMs with good chemical stability and dense microstructure. The measures of integrity test, pH, and ionic conductivity in water confirmed that the best sample is made with calcined clay at 600 °C, being similar (53% higher ionic conductivity of the eluate) or equal (integrity test and pH) to values recorded for the metakaolin-based geopolymer considered the reference material. These results were reflected in term of reticulation and morphology of samples through the analysis with scanning electron microscope (SEM) and X-ray diffraction (XRD), which show a dense and homogeneous microstructure predominantly amorphous with minor amounts of quartz, halloysite, and illite crystalline phases. Special attention was dedicated to this by-product to promote its use, given that kaolinite (and metakaolin), as primary mineral product, has a strong impact on the environment. The results obtained led us to consider this halloysite clay very interesting as an aluminosilicate precursor, and extensively deepening its properties and reactivity for the alkaline activation. In fact, the heart of this work is to study the possibility of reusing this by-product of an industrial process to obtain more sustainable high-performance binders.

## 1. Introduction

The relevance of calcined clays as sources with great potential for low-clinker blended Portland cements has significantly increased in recent decades; calcined clays being capable of lowering the compressive strength from a few point % to 13% [1]. At the same time, clay minerals have been considered potential candidates as raw materials for new alkali activated materials (AAMs) that can partly replace Portland cement [2], leading to compressive strength in the range of 25–48 MPa. They, thereby, significantly reduce CO_2_ emissions associated with Portland cement production by 70% for kaolin activated binders [3]. For workability and reactivity reasons, usually aluminosilicate powders used for the alkali-activation process are thermally activated materials [4]. The stable crystal structure of clay minerals and their platy morphology in combination with a high specific surface area, which results in a high water to binder ratio, are the cause of the low pozzolanic potential of untreated clays [5].

A common process to increase the pozzolanic activity of these clays is by thermal treatment, also called calcination (Table 1) [6]. When kaolinitic clay (type 1:1) is calcined, the pozzolanic activity is higher than that of calcined 2:1 clays, such as montmorillonite and illite [7,8,9,10]. Furthermore, there are other methods to increase the reactivity of clays described in the literature, such as mechanical activation. Mechanical activation significantly changes the structure of clay, increasing the specific surface area, and decreasing the particle size, for example from 13.89 m^2^/g to 21.1 m^2^/g and 35.7 m^2^/g, milling the clay for 4 and 8 h, respectively [11], and improving the compressive strength of paste due to the higher reactivity, also reaching 53 MP [12]. In this work the thermal treatment of clays was the subject of study. An overview of clay minerals calcination temperatures found in the literature is listed in Table 1. Different calcination temperatures are presented per each type of clay, being their variability is particularly wide. The reader interested in the temperature at which each clay reaches the highest pozzolanic index should refer to the references reported in Table 1, but we can summarize that, whereas this temperature is around 800 °C for 1:1 clay, for 2:1 clays the value increases from 900 °C to 1000 °C.

In ancient times crushed potsherds and ceramic waste material was often blended with lime to enhance the mortar’s property [23]. The first research reports regarding the use of calcined clays date to the 1950s [24] regarding the effect of calcination on natural pozzolans. In the 1960s calcined kaolinite, or metakaolin, was used in large construction units [25], but the number of studies regarding calcined clays increased significantly from the mid-1990s. The use of calcined clays in the cement industry remains limited compared to other supplementary cementitious materials (SCMs), mainly due to the complexity of clay minerals. The availability and the high number of clay resources make calcined clays rather unique in comparison to the other SCMs [6]. Finally, in 2016, a test to predict the pozzolanic activity of calcined clays with kaolinite contents ranging from 0 to 95% in a simplified system with portlandite and limestone pastes, with sulphate and alkali levels adjusted to reproduce the reaction environment of hydrating blended cements, was proposed [26].

In this work, attention is paid to a by-product of mining processes, characterized by 43–45 wt% of clay with 35% of halloysite and a minor fraction of illite. The halloysite clay, typically present in kaolin as a second mineral component, was found to have a good pozzolanic reactivity, in the range of kaolinite [18,19], as well as a positive effect on the metakaolin-based geopolymer synthesis [27]. Halloysite is a clay with identical chemical composition of kaolinite, except that halloysite may have as many as two molecules of H_2_O, as interlayer water [28]. Since kaolinitic waste clays have already been tested for their pozzolanic activity after thermal treatment [29], we also proceeded with calcination of the as-received halloysitic-illitic clayey by-product. It has already been reported that the presence of kaolinite and spheroidal halloysite exerts great influence at early ages, while tubular halloysite has a greater influence in the pozzolanic activity and the compressive strength of mortars at later ages [30]. As in our samples, the as-received clay by-product shows very low fraction of tubular halloysite, thus requiring additional activation, in particular we tested an alkaline solution of NaOH and sodium silicate on this calcined clay with the aim to consolidate a geopolymeric 3D network [31].

Geopolymers with kaolin rich in halloysite were studied and realized by many researchers to have good properties when activated with a solution of NaOH and waterglass [27]. Most studies using calcined clays in hybrid cementitious systems are limited to the utilization of metakaolin. This brings great opportunity to explore production of other alkali-activated cementitious blends with different types of clay minerals. Clay minerals are also present in other waste streams, including mining wastes [32] and dredging wastes [33]. From these various sources, clay minerals are a diverse, abundant, and widely available resource, and, therefore, have high potential as scalable precursors for cementitious materials production [2]. In this view, we inserted the aim of this research and the recovery of these clayey mine residues. Given that kaolinite (and metakaolin) has been exhaustively studied [34,35], it is considered, here, as a reference point for the halloysite clay residues under investigation. With the motivation to seek environmentally friendly and cost-effective building materials, we adopted low calcination temperatures (T max 600 °C) provided for the calcination of these clay by-product remains, which is a sustainable solution compared with the production of traditional cement materials, for which energy consumption is high, without considering that the source material of this proposed AAM is a mine residue.

In this paper we investigated the influence of the thermal treatment of the clay on the microstructure of the final products after 28 curing days. The chemical stability obtained from samples made with untreated and calcined clay, through the measures of pH and ionic conductivity in water, were investigated. The differences of reticulation of samples and the morphology of the clays were analyzed with scanning electron microscope (SEM) and X-ray diffraction (XRD). To our knowledge, there are not yet published papers on mine clay washing residues so rich in halloysite to exhibit a dehydroxylation step low enough to already be efficient in term of alkali activation at a 600 °C calcination temperature.

## 2. Materials and Methods

### 2.1. Raw Material

Mine clay washing residue, used as an aluminosilicate source for the preparation of geopolymers, is a co-product of the production of glass sands and comes from Lazio in the province of Latina in Italy. This clay is obtained following a process of separation from the sand by washing and filter pressing. The chemical composition of this mine residue was determined by the X-ray fluorescence (XRF) technique before and after calcination by using the Bruker S4 Pioneer (Billerica, MA, USA) XRF spectrometer, with accuracy of about 0.1–0.2%. Being a variable by-product, we report the compositional range in Table 2. XRD measurements were carried out with a diffractometer from Bruker/Siemens XRD, with a copper radiation of CuKα = 1.54060 Å. The data were collected between 5 and 65° 2θ angles. The step size was set to 0.02° 2θ per step. The software used in the identification of phases is Diffrac.Suite Eva with the PDF-2 database of the International Centre for Diffraction Data (ICDD), and semi-quantitative Rietveld evaluation was performed with 0.5 wt% accuracy. The range of the mineralogical composition is shown in Table 2.

**Figure 1:**
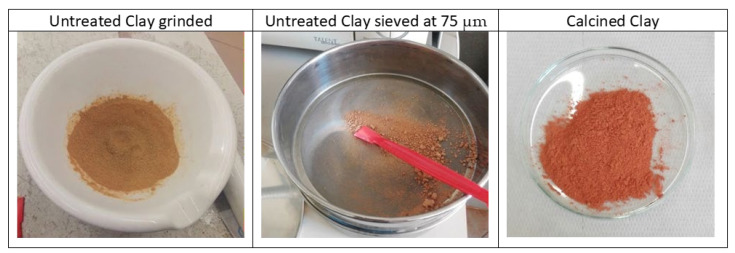
The halloysite clay before and after calcination.

**Figure 2:**
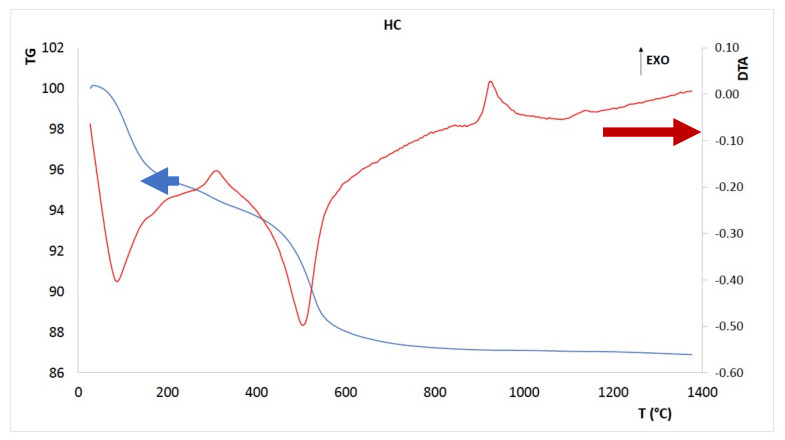
The DTA and TGA curves recorded for the as-received clay (exothermic direction is upward).

The as-received halloysite-rich clay was dried and sieved at 75 µm and then calcined at different temperatures, 450–500–600 °C, (Figure 1), based on DTA/TGA analysis (Figure 2). Thermal analysis (NETZSCH STA 409 CD, Selb, Germany) was performed with a heating rate of 10 °C/min from 25 to 1400 °C in air flushing to validate the thermal behavior of the clay and verify the temperature that is needed to guarantee full dehydroxylation of the clay minerals used for alkali activation materials. TGA results between 400 and 700 °C have been used to determine the overall content of kaolinite and halloysite, w%_K-H_, that is to say the kaolinite group minerals, using the equation [36]:

w%_K-H_ = w%_K-H hydrate__x_ × (M_K-H_)/2M_H2O_)

where w%_K-H hydrate_ indicates mass loss during the dehydroxylation of kaolinite/halloysite; M_K-H_ indicates the molecular weight of kaolinite/halloysite (Al_2_Si_2_O_5_(OH)_4_), 258.16 g.mol^−1^; and M_H2O_ is that of water, 18.02 g.mol^−1^ following the endothermic dehydroxylation to the metastable metakaolin phase that occurs in the temperature range of 450 to 700 °C according to the reaction:

Al_2_Si_2_O_5_(OH)_4_ → Al_2_Si_2_O_7_ + 2 H_2_O

The specific surface area of the clay (Table 2) was measured with the BET—Brunauer–Emmett–Teller—method with a Micromeritics Gemini V, Norcross, GA, USA, surface analyzer.

### 2.2. Alkali Activation Procedure

The alkaline activators used for sample preparation are sodium hydroxide solution, NaOH 8M, and sodium silicate solution, Na_2_SiO_3_. The sodium silicate solution was provided by Ingessil s.r.l., Verona, Italy, with a molar ratio SiO_2_:Na_2_O = 3. Sodium hydroxide solutions 8M were prepared by dissolving NaOH pellets (*Merck KGaA*, Darmstadt, Germany, purity ≥ 98%) in distilled water and stored to cool to room temperature.

Samples prepared with the clay after calcination at different temperatures (450 °C–500 °C–600 °C) were tested to evaluate the best calcination temperature to achieve a good chemical stability. All samples were prepared by mixing clay and alkaline solution (see Table 3 for formulations) for a few minutes until reaching a homogeneous paste. The paste was then poured into a plastic mold covered with plastic film and cured at room temperature for 28 days. Several formulations were tested initially with untreated and calcined clay before finding the proper molar ratio: Si/Al and Na/Al. In Table 3 are two samples, G1 and G2, with different molar ratios of Si and Na prepared with the not calcined clay. The same formulations were then prepared with the calcined clay.

### 2.3. Hardened Geopolymers Characterization

Hardened samples were removed from molds at 28 days of age. Specimens were prepared as required by each single characterization technique.

The chemical stability was defined with an integrity test in water, and by pH and ionic conductivity measurement.

The integrity test is a preliminary qualitative test to verify if the geopolymerization process has occurred. During the test a bulk specimen is immersed in distilled water with solid/liquid ratio 1:100 for 24 h. After 24 h the sample is taken out of the water and evaluated on the structural consistency of the sample, in terms of dissolution and fragmentation, and resistance of finger pressure.

Measures of pH and the ionic conductivity were performed by immersing the sample under stirring conditions at 20 ± 2 °C in deionized water with a solid/liquid ratio of 1:10 for 24 h. The measurements were carried out using electrodes immersed in the water with the sample during the 24 h period of the test, more frequently within the first two hours, and spaced during the remaining time. In this way, ionic conductivity and the pH of the eluate solutions were determined at different times (0, 5, 15, 30, 60, 120, 240, 360, and 1440 min), to obtain a trend of the change in value during the 24 h period and to have information on the amount of dissolved solid.

The pH was detected with a Hamilton type Liq-glass SL Laboratory pH sensor (Hamilton A.G., Bonaduz, Switzerland), and the electrical conductivity of the solution was measured with a calibrated cell, both of which were connected to the digital display of pH 5/6 and Ion 6- Oakton/Eutech Instruments (Oakton Instruments, Vernon Hills, IL, USA).

X-ray diffraction patterns of calcined clays, as well as of geopolymer samples, were recorded by a PW3710 diffractometer (Philips, Almelo, The Netherlands). Specimens were scanned from 5° to 70° 2 theta range on powdered samples.

Morphology observations were conducted for clay and geopolymer samples by environmental scanning electron microscopy (ESEM) using a QUANTA 200 microscope equipped with EDS (FEI, Hillsboro, OR, USA). The planar surface of the sample was placed on an Al sample holder with the silver glue, and, once dried, all the surface was vacuum-sealed golden, before being analyzed.

## 3. Results and Discussion

### 3.1. Thermal Characterization of the Clayey Co-Product

The optimal calcination conditions were determined according to the weight loss, as recorded from thermogravimetric analysis (Figure 2) similarly to the literature procedure [37]. Figure 2 presents the simultaneous thermal gravimetric and thermal analysis curves of the as-received clay. Three main peaks are visible in the DTA curve, the first two also corresponding to weight loss. Through heating the kaolinite, or halloysite raw mineral, encounters humidity evaporation at around 120–150 °C, followed by the complete elimination of the inner-surface hydroxyl groups and interlayer water molecules between 400–600 °C, thus causing shrinkage of the inter-layer distance in the c-axis direction [38]. The endothermic dehydroxylation occurs drastically in the range of 450–600 °C (compare Figure 1 in [39]), which is accompanied by an obvious loss in mass of about 4.77%, observed at temperatures below 600 °C. Using the formula to evaluate the w%_K-H_, we can confirm that the dehydroxylated amount of halloysite plus eventual traces of kaolinite is 34.1% at 600 °C. An exothermic signal is detected in the range of 850–1200 °C, with a peak at 930 °C. This exothermic process is evidence of the SiO_2_ and γ-Al_2_O_3_ phase segregation in the mineral, creating what is referred to as diphasic gels, an intermediate spinel phase [40]. The other trace exothermic signal, characteristic of kaolinite group minerals, was measured at 1150 °C, but, again, did not show any mass changes. This stage should be attributed to the emergence of a mullite phase in the mineral [41,42]. The peculiar low stability of the halloysite of this study is, once more, stressed by its early mullite crystallization with respect to literature data of the 1290 °C peak recorded for highly stable halloysite [38]. There is another trace of an exothermic peak at about 310 °C, accompanied by a slight weight loss (0.5%). This thermal event can be attributed to iron (II) hydroxides oxidation accompanied by their dehydroxylation to give hematite, according to the following reaction [43] and accounting for the increased reddish color recorded after calcination (see Figure 1):

2Fe(OH)_2_ + 1/2O_2_ → Fe_2_O_3_ + 2H_2_O

The presence of low iron-bearing phases could not be a problem, as already investigated for lateritic soils during alkaline activation [44]. In conclusion, the best results have been obtained in terms of chemical stability with calcined clay. In this case, temperature plays a fundamental role regarding the heat treatment of the starting raw material. From the thermogravimetric analysis DTA/TGA of the clay (Figure 2), there has been an extrapolated range of temperatures (400–600 °C) useful for the identification of the optimal temperature of calcination of the clay. In this temperature range we obtained a metakaolinitic phase, which is typically formed at 700 °C, or even at 800 °C, starting from kaolinite with a characteristic change in aluminum coordination from six- to four-fold [40].

### 3.2. X-Ray Diffraction of Clay after Thermal Treatment

**Figure 3:**
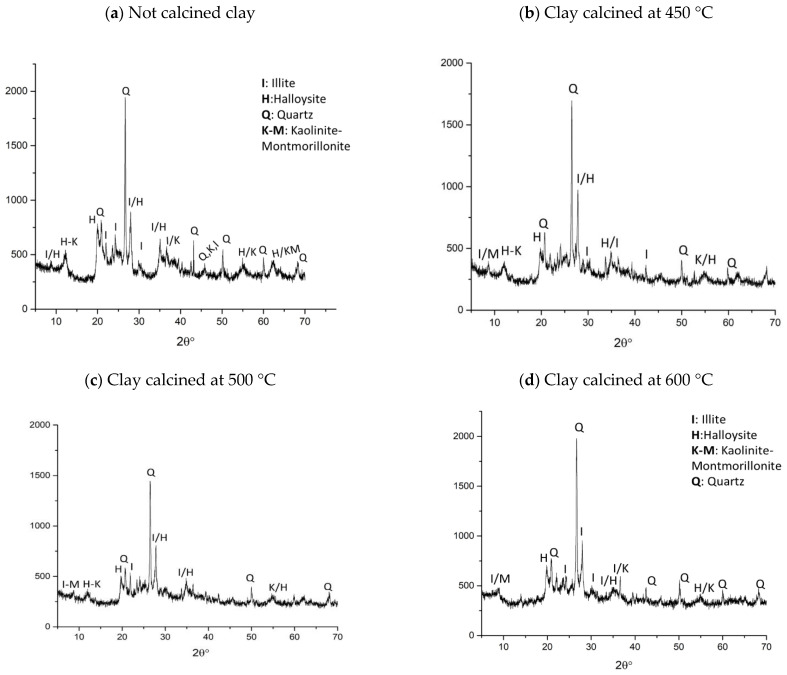
XRD patterns of clay: not calcined (**a**) and calcined at 450 °C (**b**), 500 °C (**c**), and 600 °C (**d**).

In Figure 3, the XRD analysis of clay calcined at 450 °C (b), 500 °C (c), and 600 °C (d) compared with the untreated clay (a), are reported.

The XRD pattern of the as-received clay (Figure 3a) shows the characteristic peaks of the three main minerals (quartz, illite, halloysite) listed in Table 1. Please notice the halloysite in this by-product presents the characteristic peak of 12° (corresponding to the interplanar distance of 7 Å [38]), due to the loss of the two molecules of interlayer water, lost during the drying treatment in the extraction plant. With the increase in the calcination temperature, it is possible to see the decrease in intensity of these peaks. The peak at 12° in 2θ, indicated as H-K, corresponds to the kaolinite and halloysite phase; such a peak present in pattern (a) decreases with the calcination at 450 °C in pattern (b), until it disappears in (d). Furthermore, the peaks at 35 and 62° in 2θ, typical of halloysite/illite, follow the same trend and decrease their intensity with the temperature, with illite being still visible at about 8° in 2θ at 600 °C in Figure 3d [45]. The amorphous phase also increases in the large halo from 20 to 35 °C, being more visible at 600 °C, confirming the typical disordered structure of metakaolin prone to the alkali activation. Once more we can confirm the calcination products (at the different temperatures) resulted in a mineralogically more unstable phase than the as-received precursors promising to be more reactive during alkali activation.

### 3.3. Chemical Stability of the Alkaline Activated Materials

**Figure 4:**
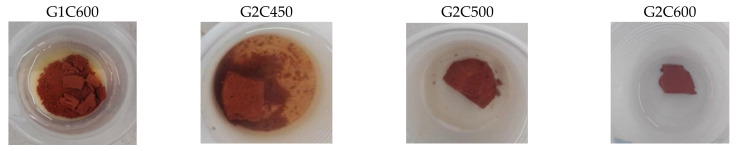
Integrity test in water after 24 h of samples G1C600, G2C450, G2C500, and G2C600.

Table 3 shows the formulations for samples G1 and G2 prepared with the non-calcined clay. These samples do not resist in water after the 24 h of immersion, as prescribed by the integrity test. This result confirms that the reticulation process did not take place since the pozzolanicity of the as-received clay is too low to be activated at room temperature [46]. When the same formulations were tested with calcined clay at different temperatures, it was noted that, as the calcination temperature and time increased, the consolidated samples appeared more stable, and with better final properties. Figure 4 shows the photos of the samples with calcined clay after immersion in water for 24 h. Samples G1C600 and G2C450 were dissolved in water, losing the structural consolidation. Samples G2C500 and G2C600 showed improvements in terms of chemical stability in water; they remained solid after the 24 h of immersion and the color of the water remains clear, in particular for G2C600. Better results were achieved also in terms of workability since for this set it was not necessary to add any amount of water to the mixture of powder and alkaline solution. The best results were obtained for the formulation G2 made with heat-treated clay at 600 °C for 2 h, with NaOH 8M and Na_2_SiO_3_ in 1:1 volume ratio.

**Figure 5:**
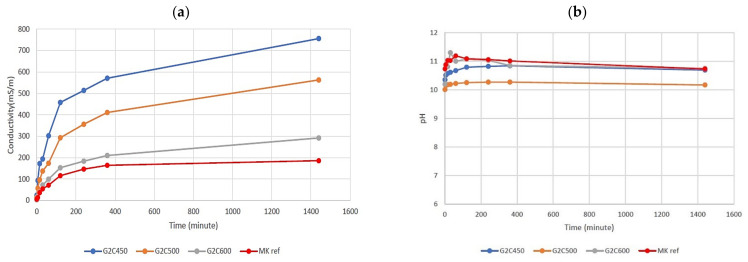
Results of ionic conductivity (**a**) and pH (**b**), respectively, for eluates from samples G2C600, G2C500, and G2C450. Geopolymers produced with commercial metakaolin were used as reference materials data from Ref. [51].

The pH and ionic conductivity measurements performed on the eluate after the immersion of the sample under stirring conditions at 20 ± 2 °C in deionized water with a solid/liquid ratio of 1/10 for 24 h, confirmed the higher stability of the sample with the clay calcined at 600 °C (Figure 5). It is clearly visible that ionic conductivity decreases with the increment of the calcination temperature, due to the lower release of ions in water and the higher chemical stability of the sample. This behavior is typical of these materials, and it was reported in a previous work [47]. Figure 5 shows the values of pH, which remain constant between 10.2 and 11 for the duration of the test for all samples, according to the results observed in the literature for MK-based geopolymers [48,49].

Figure 5 shows, in red, the MK reference values of pH and ionic conductivity, to compare with the other samples [50]. The materials obtained with the calcination of this clayey by-product at 600 °C are comparable with reference MK-based geopolymers. Indeed, the values of conductivity of G2C600 reported are 292 mS/m compared with 190 mS/m of MK-based geopolymer, while the pH results are approximately the same. Instead, the values of G2C450 and G2C600 of conductivity are approximately 2 or 3 times higher than the values of the MK-based geopolymers standard.

### 3.4. SEM and XRD of Clay and Geopolymer

The best temperature of calcination of the clay (600 °C) and geopolymer made with this clayey by-product (G2C600) was analyzed with the scanning electron microscope (SEM) compared with the clay and geopolymer without heat treatment.

**Figure 6:**
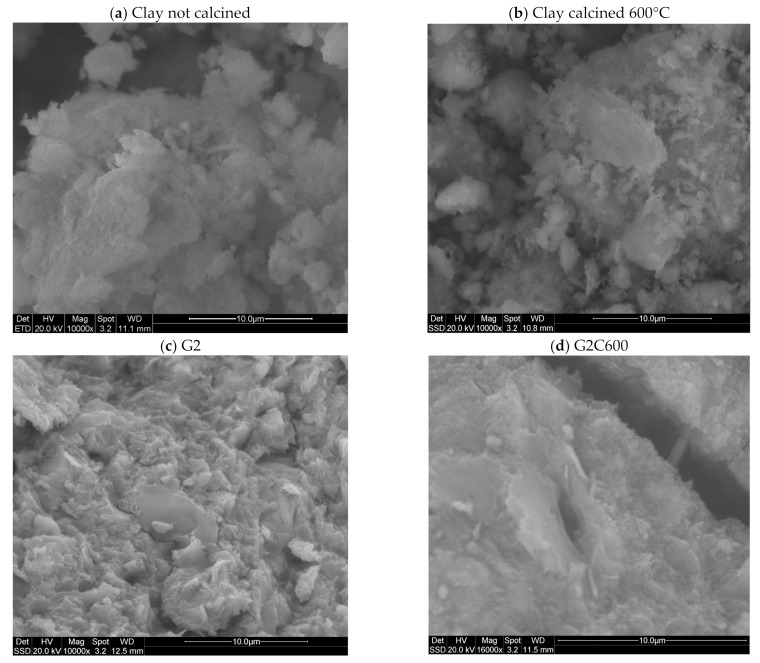
SEM images of the as-received clay by-product (**a**), calcined powder at 600 °C (**b**), fractured surface of sample G2 (**c**), and G2C600 (**d**).

Figure 6 shows the SEM micrographs of the non-calcined clay (a) and after calcination (b), and their corresponding alkali activated materials: G2 and G2C600. The clayey platelets before and after calcination do not change their morphology, indicating that the calcination temperature was not sufficient to produce strong aggregated nor sintering. This very loose aspect of the aluminosilicate powder is propaedeutic to its high and efficient dispersion within the alkali activated paste, as well as to a good workability. Please notice the absence of tubular halloysite crystals, which are not characteristic of these mine residues. After alkali activation, the microstructure of G2C600 presents a very compact morphology with a flatter fractured surface with respect to G2, indicating higher mechanical performance. The last property is also accompanied by the higher chemical stability as deduced from pH and ionic conductivity tests.

**Figure 7:**
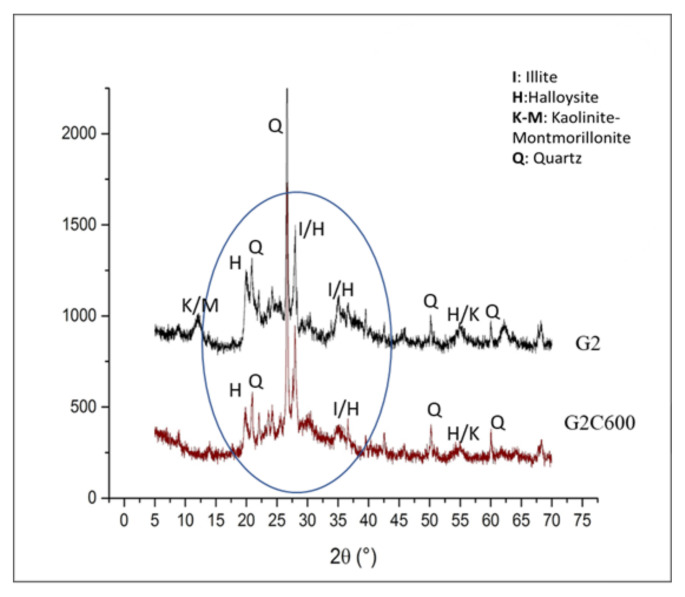
XRD patterns of G2 and G2C600.

Comparing the XRD patterns of G2 and G2C600 in Figure 7, it is possible to see a difference between these two formulations in the range 15 and 35° in 2θ (circled area). The amorphous band in the spectrum of G2C600 in this range is more pronounced than in G2, denoting a higher amount of amorphous gel correlated to a better reticulation of samples during the geopolymerization process. This last observation is coherent with the examples reported in the literature for a typical AAM amorphous pattern indicative of a geopolymeric gel [51,52]. Therefore, it can be affirmed that the investigated clay by-product requires at least 600 °C calcination temperature to reach an efficient consolidation in an alkaline environment.

## 4. Conclusions

In this study, the influence of thermal treatment of the clay by-product, in terms of its effects on the microstructure of the samples obtained after alkali activation, has been investigated.

The results, in terms of chemical stability of ionic conductivity and resistance in the aqueous environment, showed that the calcination of this clayey by-product is necessary to obtain a good reticulation and alkali activation of the material. The optimum temperature of calcination should not be lower than 600 °C, reaching a performance similar to that of traditional metakaolin based geopolymers. The results of the integrity test in water showed a better resistance of the sample G2C600. Additionally, the values of pH and ionic conductivity of the sample G2C600 are comparable with the reference values of the MK based geopolymer.

These results in chemical stability were confirmed by X-ray diffraction spectra of samples, which clarified the good reticulation of samples with calcined clay (G2C600), compared to samples made with clay without heat treatment (G2). The band between 15 and 35° in 2θ of the spectra of G2C600 showed a typical amorphous pattern of a geopolymeric material.

The SEM analysis confirmed the formation of a compact geopolymeric gel leading us to predict good mechanical performances. After alkali activation, the microstructure of G2C600 presents a very dense and compact morphology with a flatter fractured surface compared to G2, while the clayey platelets before and after calcination presented similar morphology, suggesting that the calcination temperature was not sufficient to produce strong aggregates.

This work is one of the many that suggest the use of industrial by-products for the production of new binders with a better environmental impact. The waste clay presented in this work is a co-product obtained from the washing and filtering operations in the production cycle of different grades of sands from a single mine. The valorization and exploitation of this clayey residue (43–45 wt% of clay), if employed for the manufacturing of new materials as alkali activated binders, could become a resource for a new sustainable production. Additionally, the room temperature consolidation, via alkaline activation of low cost Na-bearing solutions, is also proposed to keep costs in line with cheap building materials. Apart from the aqueous solution, the proposed technology has no water consumption. Processing the proposed materials does not, in turn, generate waste, thus closing the loop of the green economy circle with a no water and no waste manufacturing proposal.

In future studies, the mix-design of these simple binders can be engineered and optimized, either in the direction of a mechanically performant material (including fillers and reinforcements), or in the development of a porous material (including poring agent and surfactants). We can propose our formulations in place of metakaolin-based geopolymers as matrices for more complex composite materials, meeting several performance requirements in terms of engineering, architectural, cost, and eco-efficiency, etc.

## Figures and Tables

**Table 1:** Overview of the calcination temperatures for different clay types reported in the literature.

Clay Type	Calcination T (°C)	Reference
Kaolinite	550–950	[13]
	650–800	[14]
	600–800	[7]
	500–700	[15]
	600–800	[16]
	650	[17]
Halloysite	450–700	[18]
	600–750	[19]
Kaolinite/halloysite	600–800	[1]
	600–700	[20]
Illite	650–930	[13]
	600–800	[7]
	600–800	[16]
	600–800	[1]
Illite/Montmorillonite	730–920	[13]
	600–800	[21]
	600–800	[7]
	500–800	[15]
	600–800	[16]
Mica clay	560–960	[13]
	700–1100	[22]
	600–800	[1]

**Table 2:** Chemical (XRF) and mineralogical (XRD) compositional range of clay (* Iron Oxides/hydroxides expressed as F_2_O_3_).

Oxide Composition	Clay	Phase	Clay
SiO_2_(wt%)	51–55	Quartz(wt%)	22–24
Al_2_O_3_ (wt%)	26–30	Alkali feldspar (wt%)	10
Fe_2_O_3_ * (wt%)	4	Illite (wt%)	8–10
TiO_2_ (wt%)	≤1	Illite/smectite (wt%)	1–2
CaO (wt%)	≤0.5	Plagioclase (wt%)	22–23
MgO (wt%)	≤1	Halloysite (wt%)	35
K_2_O (wt%)	≤2	Goethite (wt%)	1–2
Na_2_O (wt%)	≤2		
		D90 (µm)	18–20
LOI (wt%)	10	B.E.T. (m^2^/g)	35.90 ± 0.22

**Table 3:** Formulations of the samples.

Sample	Calcination of Clay		S/L	Si/Al	Na/Al	Alkaline Solution
	(°C)	(h)				
G1	-	-	1	2.3	1.33	NaOH 8M+ Na_2_SiO_3_
G2	-	-	1.46	2.04	2.12	NaOH 8M+ Na_2_SiO_3_
G2C600	600 °C	2	1.66	2.04	2.12	NaOH 8M+ Na_2_SiO_3_
G2C500	500 °C	2	1.66	2.04	2.12	NaOH 8M+ Na_2_SiO_3_
G2C450	450 °C	2	1.66	2.04	2.12	NaOH 8M+ Na_2_SiO_3_
G1C450	600 °C	2	1.06	2.3	1.33	NaOH 8M+ Na_2_SiO_3_
G1C500	500 °C	2	1.06	2.3	1.33	NaOH 8M+ Na_2_SiO_3_
G1C600	450 °C	2	1.06	2.3	1.33	NaOH 8M+ Na_2_SiO_3_
